# Mechanistic Insight on the Interaction between OPN and Integrin *ανβ*3 in Osteoarthritis

**DOI:** 10.1155/2020/2905634

**Published:** 2020-10-20

**Authors:** Yuhao Yuan, Qing Liu, Ziyi Wu, Wei Luo

**Affiliations:** ^1^Department of Orthopaedics, Xiangya Hospital, Central South University, Changsha, China; ^2^Department of Spine Surgery, The Third Xiangya Hospital, Central South University, Changsha, China

## Abstract

Osteoarthritis (OA) is a joint disease characterized by cartilage degeneration. Osteopontin (OPN) is involved in the initiation, repair, and maintenance of metabolic homeostasis in normal articular cartilage. This study investigated the role of OPN and its interaction with the integrin *ανβ*3 receptor in the expression of hyaluronic acid (HA) in OA chondrocytes. Overexpression of OPN significantly increased the expression of integrin *ανβ*3 and hyaluronic acid synthases (HAS) and synthesis of HA. Depleting OPN in OA chondrocytes showed the opposite trend for integrin *alpha;νβ*3, HAS, and HA. Nonspecifically and specifically blocking integrin receptor using GRGDSP and integrin *ανβ*3 antibody downregulated HAS and HA; both were inhibited to similar extents. The expression of HAS and HA was predominantly regulated by the interaction between OPN and integrin *ανβ*3. Taken together, we have delineated the importance of the OPN/integrin *ανβ*3/HAS/HA axis in OA and identified OPN as a promising candidate for molecular therapy for use in patients with OA.

## 1. Introduction

Osteoarthritis (OA) is a joint disease characterized by articular cartilage degeneration, subchondral sclerosis, and synovitis. The knee joint is the most common site for OA. OA has a high rate of incidence, especially in older women. Elderly above the age of 60 and 75 are associated with a rate of prevalence of 50% and 80%, respectively, and the disability rate is up to 53% [1]. The primary symptoms of OA include joint pain, limited movement, and deformity. Thus, OA lowers the patient's health and quality of life and is a socioeconomic burden [2]. Degeneration of the articular cartilage is pivotal in the development of OA [3, 4]. Chondrocytes are responsible for the synthesis and degradation of extracellular matrix and play a role in tissue homeostasis [5]. Apoptosis and destruction of chondrocytes comprise the main pathological changes during the early stages of inflammation in individuals with OA.

Osteopontin (OPN) is a negatively charged, noncollagen, bone matrix glycoprotein widely distributed in the extracellular matrix, bone tissue, and inflammatory sites [6]. OPN interacts with receptors, such as integrin and CD44, to regulate inflammation, immunity, bone metabolism, and tumor metastasis among other physiological and pathological processes [7]. Previous studies have shown that OPN is involved in the occurrence, repair, and maintenance of metabolic homeostasis of normal articular cartilage, suggesting a protective role of OPN in the pathological development of OA by regulating the components of the extracellular matrix of the articular cartilage [8, 9]. Therefore, it is imperative to understand the difference in expression of OPN and its downstream regulatory molecules in the cartilage of individuals with OA to develop OPN as a novel therapeutic for OA.

Integrin *ανβ*3 is a transmembrane heterodimer glycoprotein that is the predominant family of cell surface receptors [10, 11]. Studies have shown that integrin *ανβ*3 plays an extremely important role in signal transduction, differentiation, and proliferation in chondrocytes [12–14]. Integrin *ανβ*3 levels vary with the degree of degeneration in the cartilage of individual with OA [10]. The interaction between OPN and integrin *ανβ*3 ligand receptor mediates the adhesion of vascular smooth muscle cells and enhances tumor cell invasion [7]. In the process of tumor cell migration, integrin *ανβ*3 also interacts with hyaluronic acid (HA) to mediate the matrix production of tumor cells, improve adhesion, and regulate differentiation and metastasis of tumor cells [12, 15].

HA is a glucosamine-containing polysaccharide and the main component of the extracellular matrix. It functions in joint lubrication, anti-inflammation, cartilage protection, and balance in cartilage matrix [16] and is crucial in maintaining the normal function of joints. HA has been widely used to control the development of OA. HA synthases (HAS1, HAS2, and HAS3) induce the production of HA and are secreted by chondrocytes [17]. The molecular weight of HA in synovial fluid from patients with OA and rheumatoid arthritis is lower than the molecular weight of that from healthy individuals [18]. The serum HA content is an important biomarker for radiologic OA [19]. OPN and HA are involved in the pathogenesis of OA and other diseases [20].

We hypothesized that OPN binds to integrin *ανβ*3 on chondrocytes from individuals with OA; this regulates the secretion of HA by regulating the expression of HAS and participates in the pathogenesis of OA. Therefore, the primary objective of the present study was to decipher the role of OPN and its interaction with the integrin *ανβ*3 receptor in the expression of HA in OA chondrocytes.

## 2. Material and Methods

### 2.1. Extraction and Culture of Chondrocytes

Cartilage tissues were collected from individuals with OA (Kellgren-Lawrence III-IV grade) during artificial surface knee arthroplasty at the Department of Orthopedics, Xiangya Hospital, Central South University. Normal articular cartilage was obtained from patients with malignant bone tumors who underwent segmental resection and hinge knee joint replacement. Chondrocyte filtrates were isolated as previously described [5]. Filtrates were cultured in DMEM-F12 complete culture medium (Gibco, Burlington, ON) supplemented with 10% fetal bovine serum (Gibco, USA). The chondrocytes were divided into 25 mm^2^ culture flasks at a cell count of 1 × 10^5^ cells/ml and cultured at 37°C in a 5% CO_2_ incubator.

### 2.2. Cartilage Staining and Proliferation Assay

The cartilage tissues were washed twice with phosphate-buffered saline (PBS) followed by fixing in 4% paraformaldehyde, decalcified in 10% ethylenediaminetetraacetic acid, washed with distilled water, stained with toluidine blue and alizarin red, destained with gradient alcohol, and observed under the microscope after drying. The isolated chondrocytes were also stained using toluidine blue and alizarin red. The cell counting kit-8 was used to detect chondrocyte proliferation. The cell suspension was inoculated into 96 well plates with 100 *μ*l/well at a count of 2,000 cells/well. At the corresponding time points, we added 10 *μ*l of the CCK-8 solution (Beyotime, Hangzhou, China) to the wells, incubated the plates for 2 h, and measured absorbance at 450 nm.

### 2.3. Immunohistochemistry

Formalin-fixed paraffin-embedded cartilage samples were sectioned to 4 *μ*m in thickness, dewaxed using xylene, hydrated with alcohol, and incubated with 3% hydrogen peroxide at room temperature for 25 min. PBS containing serum was used to block the nonspecific antigens. The diluted antibody targeting OPN (D121078, BBI Life Sciences, China) was incubated with the sections overnight at 4°C. Subsequently, we incubated the sections with secondary antibody (AS064, AB clonal, China), stained, and visualized.

### 2.4. Immunofluorescence (IF)

Chondrocytes were washed thrice with PBS at 4°C and fixed in 4% paraformaldehyde for 15 min. Subsequently, chondrocytes were washed thrice with PBS containing 0.1% Triton X-100 and PBS each for 5 min. The cells were incubated overnight with primary antibodies against integrin *ανβ*3 (KT200225, Fusheng, Shanghai, China) and HAS1 (A-AO1427a, Amylet, Wuhan, China) at 4°C in a wet box followed by fluorescent secondary antibody for 1 h. The samples were incubated with DAPI (Servicebio, Wuhan, China) in the dark for 3 min. Excess DAPI was washed out using PBS containing Triton X-100. Proteins were visualized using the Leica subduction-sp8 imaging microscope.

### 2.5. Immunoblotting

Immunoblotting analyzed the expression of proteins with the primary antibodies used in IF and immunohistochemistry as previously described [15, 17], Cell lysates (20 *μ*g) were subjected to 10% sodium dodecyl sulfate-polyacrylamide gel electrophoresis. The separated proteins were transferred to the nitrocellulose membrane. The membranes were blocked with triple-buffered saline containing skimmed milk and Tween 20 (0.1%), incubated with secondary antibody (AS064, AB clonal, China), and visualized using chemiluminescence.

### 2.6. Cell Transfection and Receptor Blocking

After the OA chondrocytes were evenly covered with 6-well plates, they were randomly divided into 5 groups: si-OPN group—chondrocytes were transfected with a plasmid containing the siRNA against OPN (RiboBio, Guangzhou, China) as per instructions provided to deplete cells of OPN. Dilute the transfection reagent (Lipofectamine 3000) with serum-free medium, mix well, prepare liposome-DNA mixture and liposome-siRNA mixture, and incubate in dark at room temperature for 1 h. After that, the liposome DNA complex and liposome siRNA mixture were added into OA chondrocytes with equal volume. The cells were incubated in a 5% CO_2_ incubator at 37°C for 2 days and then take them out for experimentation; OA-NC group—OA chondrocytes were transfected with plasmid without silencing sequence as control, and the specific method is the same as the si-OPN group; rhOPN group—1 *μ*g/ml recombinant human osteopontin (rhOPN) (Genechem, Shanghai, China) was added to the OA chondrocyte culture medium to upregulate the expression of OPN in the culture system. After intervention for 24 hours, the expression of related proteins and RNA was detected; *ανβ*3 Ab+rhOPN group—pretreated with anti-integrin *ανβ*3 blocking mAb (100 *μ*g/ml) (593274-97-6, Baileibo, Beijing, China) to interfere with chondrocytes for 1 h to block the binding of OPN-integrin *ανβ*3 and then add 1 *μ*g/ml rhOPN intervention for 23 h; GRGDSP+rhOPN group—GRGDSP (91037-75-1, MCE, China) peptide (100 *μ*g/ml) was used to block the binding of OPN to integrin subtype receptors for 1 h, and then 1 *μ*g/ml rhOPN was added for 23 h.

### 2.7. Quantitative Real-Time Polymerase Chain Reaction (RT-qPCR)

As previously described [17, 21], total RNA from chondrocytes and cartilage tissues was extracted using TRIzol (CW Biotech, Beijing, China). RT-qPCR was performed after reverse transcription as per the protocol provided with the miRNA reverse transcription kit (TaKaRa, Japan). *β*-Actin levels were used as the internal reference. Relative miRNA expression was calculated using the 2^−ΔΔCt^ method.  Table 1 lists the sequences for the used.

### 2.8. Enzyme-Linked Immunosorbent Assay (ELISA)

We used the HA detection kit (CUSABIO, Wuhan, China) based on a solid-phase sandwich ELISA to detect HA levels. The purified antibody was used to coat the microporous plate. We added the standard, sample, biotin, and enzyme together to the antibody-coated plate to initiate the thermophilic reaction to ensure complete binding to micropore wall. After incubating, the unbound components were washed out, and substrate was added to detect antigen-antibody interaction. Readout was based on the yellow color developed. Finally, absorbance was measured to calculate sample concentration (Multiscan MK3, Thermo, USA).

### 2.9. Statistical Methods

The GraphPad Prism software was used for statistical analysis. All experiments were repeated at least three times independently. The experimental results were expressed as mean ± standard deviation, and *t*-test was used for comparison between groups. *P* < 0.05 was statistically significant.

## 3. Results

### 3.1. OPN, Integrin *ανβ*3, HAS, and HA Are Overexpressed in Chondrocytes from Individuals with OA

**Figure 1:**
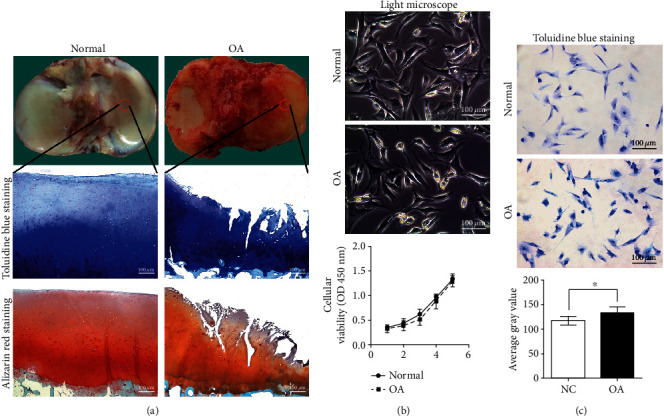
Staining and identification of cartilage tissue and cells. (a) Toluidine blue and alizarin red staining showed multiple rough and uneven cracks that extended to the calcified layer in the cartilage from individuals with osteoarthritis (OA). There was a significant reduction in the extracellular matrix. (b) OA and normal chondrocytes proliferated to same extent. (c) Mucopolysaccharide content and calcium precipitation were significantly higher in OA chondrocytes than those in normal chondrocytes. ^∗^*P* < 0.05, ^∗∗^*P* < 0.01, ^∗∗∗^*P* < 0.001.

**Figure 2:**
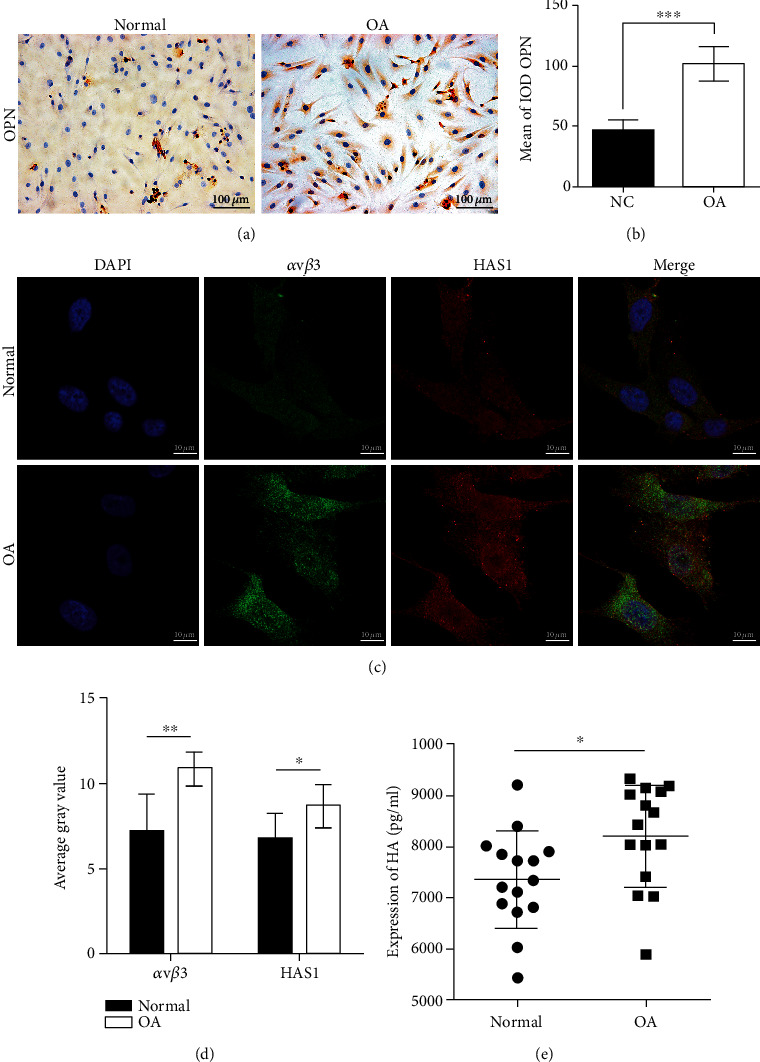
Expression of OPN, integrin *ανβ*3, and HAS1 and secretion of HA in the OA cartilage. (a, b) Immunohistochemistry showed that OPN was overexpressed in the OA cartilage (*P* < 0.001). (c, d) Immunofluorescence showed overexpression of integrin *ανβ*3 in OA chondrocytes (*P* < 0.01) and cytoplasmic and cell membrane localization. HAS1 levels were also higher in the OA chondrocytes (*P* < 0.05) and localized to the cytoplasm. (e) Enzyme-linked immunosorbent assay showed that OA chondrocytes highly expressed HA (*P* < 0.05). ^∗^*P* < 0.05, ^∗∗^*P* < 0.01, ^∗∗∗^*P* < 0.001.

**Figure 3:**
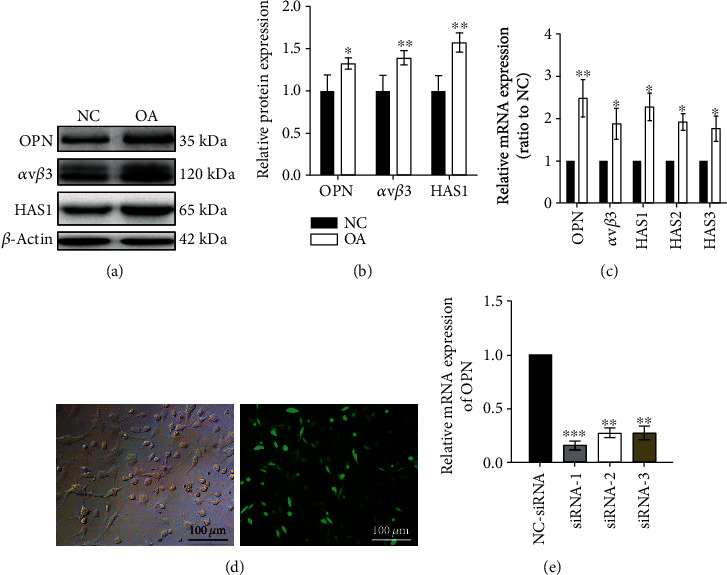
Protein and mRNA levels of OPN, integrin *ανβ*3, and HAS in OA chondrocytes. (a, b) OA chondrocytes showed increased protein levels of OPN, integrin *ανβ*3, and HAS. Immunoblots also showed binding between OPN and integrin *ανβ*3. (c) The mRNA levels of OPN, integrin *ανβ*3, and HAS were also higher in the OA chondrocytes. (d, e) We generated three plasmids bearing the siRNA against OPN. Although we observed similar rates of OPN depletion, we selected siRNA-1 for the subsequent experiments. ^∗^*P* < 0.05, ^∗∗^*P* < 0.01, ^∗∗∗^*P* < 0.001.

Toluidine blue and alizarin red staining showed that, compared to healthy cartilage, there were multiple cracks on the surface of cartilage tissues in individuals with OA (OA cartilage). The cracks expanded to the calcified layer of cartilage, number of chondrocytes significantly reduced, and diminished extracellular matrix (Figure 1(a)). The glycosaminoglycan content in OA chondrocytes was significantly higher than that in normal chondrocytes (*P* < 0.05; Figure 1(c)), but there was no significant difference in the proliferation of OA and normal chondrocytes (Figure 1(b)). Immunohistochemistry revealed that, compared to healthy cartilage tissue, OPN was significantly higher in OA cartilage tissue (*P* < 0.001; Figures 2(a) and 2(b)). IF showed that integrin *ανβ*3 was overexpressed in OA cartilage chondrocytes (*P* < 0.01) and primarily localized to the cytoplasm and cell membrane. HAS1 was also overexpressed in OA chondrocytes (*P* < 0.05) and localized to the cytoplasm (Figures 2(c) and 2(d)). ELISA demonstrated that OA chondrocytes secrete a high content of HA (Figure 2(e)). Moreover, compared to normal chondrocytes, OA chondrocytes exhibited higher mRNA and protein levels of OPN, integrin *ανβ*3, and HAS (Figures 3(a)–3(c)). We also observed binding between OPN and integrin *ανβ*3 (Figure 3(a)).

### 3.2. Integrin *ανβ*3 Expression Correlates with That of OPN

**Figure 4:**
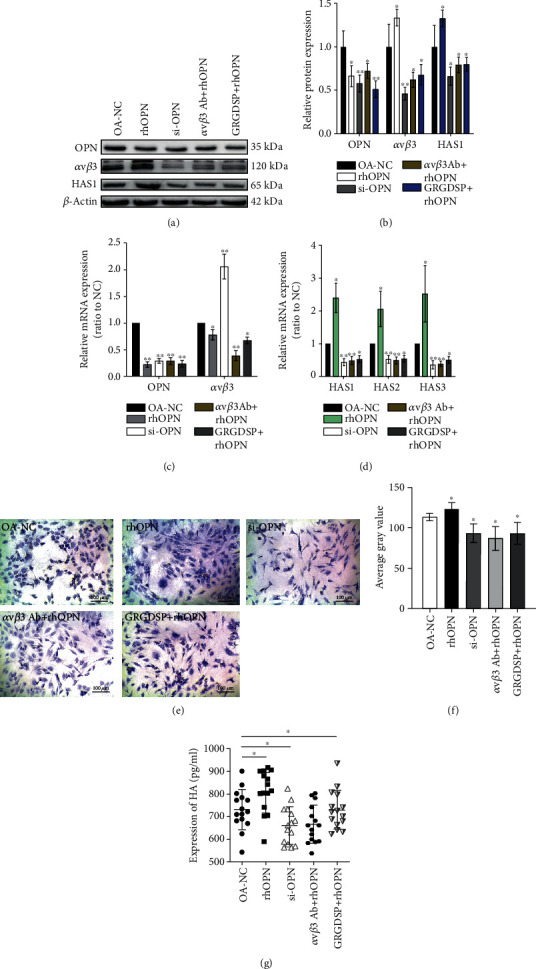
Effect of overexpression and knockdown of OPN. (a, b) rhOPN-mediated overexpression of OPN increased the protein levels of integrin *ανβ*3 and HAS1 in OA chondrocytes. Depleting chondrocytes of OPN decreased the protein levels of integrin *ανβ*3 and HAS1 in OA chondrocytes. Treating OPN-overexpressing OA chondrocytes with GRGDSP significantly reduced the protein levels of integrin *ανβ*3 and HAS1. However, using the antibody specific for integrin *ανβ*3 in OPN-overexpressing OA chondrocytes downregulated integrin *ανβ*3 and HAS1. (c, d) From the mRNA level, the expression of integrin *ανβ*3 was downregulated in the rhOPN group and upregulated in the si-OPN group. The mRNA levels of HAS1, HAS2, and HAS3 positively correlated with the expression of OPN. The mRNA levels of HAS1, HAS2, HAS3, and integrin *ανβ*3 decreased upon blocking the binding between OPN and integrin receptor. (e, f) Toluidine blue staining showed that the content of glycosaminoglycan increased in rhOPN group, but decreased significantly in si-OPN, GRGDSP+rhOPN, and integrin *ανβ*3+rhOPN groups. (g) Enzyme-linked immunosorbent assay showed that the OA chondrocytes secreted HA commensurate with the changes in HAS expression. ^∗^*P* < 0.05, ^∗∗^*P* < 0.01, ^∗∗∗^*P* < 0.001.

Comparing the OA-NC, rhOPN, and si-OPN chondrocytes, we confirmed the rhOPN-mediated overexpression of integrin *ανβ*3. Silencing OPN in OA chondrocytes reduced the protein levels of integrin *ανβ*3 (Figures 4(a) and 4(b)). The binding relationship between OPN and integrin *ανβ*3 can also be found in strip development. Using RT-qPCR, we observed a decrease and increase in the mRNA levels of integrin *ανβ*3 in the rhOPN and si-OPN groups, respectively. This was contrary to the protein levels (Figure 4(c)). Thus, integrin *ανβ*3 expression was regulated by OPN.

### 3.3. The Expression of HAS and HA Is Also Regulated by OPN

Compared with the OA-NC group, the protein levels of HAS1 in OA chondrocytes increased significantly in the rhOPN-transfected cells, but decreased upon the depletion of OPN (Figures 4(a) and 4(b)). Moreover, the mRNA levels of HAS1, HAS2, and HAS3 were the highest in cells overexpressing OPN and lowest in cells depleted of OPN (Figure 4(d)). This was consistent with the trend observed by immunoblotting. Toluidine blue staining showed that the glycosaminoglycans in the rhOPN group were increased, while the glycosaminoglycans content in the si-OPN group was significantly reduced, which indirectly reflects that the expression of HA is regulated by OPN (Figures 4(e) and 4(f)). ELISA confirmed the increased and decreased secretion of HA in the rhOPN and si-OPN groups, respectively (Figure 4(g)). This indicated that OPN regulates HAS expression and HA secretion.

### 3.4. OPN Affects the Secretion of HA upon Interacting with Integrin *ανβ*3

In GRGDSP+rhOPN OA chondrocytes (with inaccessible integrin-related receptor), there was a decrease in the protein and mRNA levels of HAS1 and integrin *ανβ*3. Specifically blocking the integrin *ανβ*3 receptor in the *ανβ*3 Ab+rhOPN OA chondrocytes, the protein and mRNA levels of HAS1 and integrin *ανβ*3 reduced significantly (Figures 4(a)–4(d)). The rates of inhibition of HAS1 expression in the two groups were similar, and in the integrin-related receptor combined with OPN, the subtype of *ανβ*3 was dominant (Figures 4(b) and 4(c)). The mRNA levels of HAS2 and HAS3 also decreased once the interaction between OPN and integrin *ανβ*3 was inhibited (Figure 4(d)). Staining analysis showed that the glycosaminoglycans were significantly reduced in the GRGDSP+rhOPN and integer *ανβ*3+rhOPN groups(Figures 4(e) and 4(f)). HA secretion in each group was consistent with the expression of HAS (Figure 4(g)). Therefore, OPN may promote the expression of HAS in OA chondrocytes, and OPN may regulate the expression of HAS via that of integrin *ανβ*3 to promote HA secretion.

## 4. Discussion

In this study, we demonstrated that (1) the surface of the articular cartilage was severely worn out, there was a significant reduction in cartilage matrix components, and calcium deposition was reduced; (2) compared to normal chondrocytes, chondrocytes from individuals with OA overexpressed OPN, integrin *ανβ*3, and HAS and secreted more HA; (3) modulating the levels of OPN revealed a positive correlation between the expression and secretion of integrin *ανβ*3, HAS, and HA with the levels of OPN; and (4) blocking the RGD fragments of the common domain of integrin receptors (including *ανβ*3, *ανβ*1, *ανβ*5, *ανβ*6, and *α*5*β*1) [22, 23] using GRGDSP showed reduced expression and secretion of HAS and HA, respectively. Moreover, specifically blocking integrin *ανβ*3 efficiently inhibited the interaction between OPN and integrin *ανβ*3 and reduced the expression and secretion of HAS and HA. Meanwhile, we also observed that the protein and mRNA expression of OPN were downregulated in the experimental group with rhOPN intervention (including the rhOPN group, *ανβ*3Ab+rhOPN group, and GRGDSP+rhOPN group). We consider that this is because the addition of rhOPN through the extracellular culture environment will have a negative feedback effect on the expression of OPN in cells. Therefore, the expression of OPN in the results of immunoblotting and RT-qPCR is reduced, but its overall effect on downstream molecules of OPN is the same, respectively. Data from this study confirmed our previous report and showed the correlation between OPN, integrin *ανβ*3, HAS, and HA in OA. We have provided a mechanistic insight into the pathogenesis of OA and identified a novel therapeutic target.

OPN has a wide range of biological functions, such as bone mineralization, tumor cell metastasis, inflammation, and immune response [24–26]. Numerous studies have investigated OPN expression in OA [27]. Li et al. [9] reported that OPN affects the pathogenesis of OA by regulating the level of macrophage-degrading enzyme. Wang et al. [28] showed that OPN regulates synovial cell proliferation in OA. The degree of expression positively correlated with the degree of cartilage degeneration. OPN also plays an important role in the development, repair, and homeostasis of normal articular cartilage [7]. However, Gao et al. [8] demonstrated the high expression of OPN in the knee cartilage and synovial tissue of individuals with OA. Li et al. [9] suggested that OPN can increase the apoptosis of OA chondrocytes by regulating the level of degradation enzymes in M1 macrophages; these results seem to be contrary to our experimental results that OPN plays a protective role in OA. But we believe that the secretion of OPN increases with the aggravation of arthritis, because the inflammatory factors stimulate the body to cause the protective high expression of OPN, and the enhancement of the apoptosis of M1 macrophages is actually to remove the degenerated chondrocytes in the joints, which may be beneficial to the body as a whole. Perhaps OPN may play a positive role and a negative role in the overall pathogenesis of OA, but in general, which part of the effect is greater still needs further research. In this study, we observed the overexpression of OPN in OA chondrocytes and interaction between OPN and integrin receptors to mediate downstream effects.

Many subtypes of integrin receptors bind with OPN. This interaction regulates the expression of tumor-related proteins during carcinogenesis in patients with rectal and/or lung cancer [29, 30]. Moreover, integrin interacts with HA to modulate differentiation and metastasis of tumor cells [31, 32], suggesting a mechanism involving the stimulation of downstream effects upon OPN and integrin receptor interaction. This includes extracellular signaling and regulation of HA synthesis, thereby affecting the development of OA. The peptides containing GRGDSP fragments used in this experiment contain RGD sites that can bind to the cell surface integrin *ανβ*3, *ανβ*1, *ανβ*5, *ανβ*6, and *α*5*β*1. By competitively binding with OPN RGD sequence, the specific antibodies can block the RGD sequence required for interaction between the integrin and OPN. This significantly reduced the expression of HAS and synthesis of HA. A recent study showed that integrin *ανβ*3 was overexpressed on the surface of osteoclasts, various tumor cells, and vascular endothelial cells [33]. Integrin *ανβ*3 comprises the main integrin that promotes the adhesion of cells to the extracellular matrix, mediates integrin signaling, and participates in a variety of pathological processes [34, 35]. We considered integrin *ανβ*3 as one of the main receptors for OPN and blocked the binding between OPN and integrin *ανβ*3. Thus, OPN may serve as a promising therapeutic target for OA.

We did not observe a complete shut-off of HA synthesis after blocking the ligand receptor interaction. This could be attributed to the following reasons. First, HA is an important component in chondrocytes. It is involved in signal transduction with integrin *ανβ*3 and is regulated by other signal transduction molecules. For example, integrin *α*5*β*1 and HA interact to enhance tumor cell invasion [32]. Second, OPN regulates the expression of HA via integrin *ανβ*3 and participates in extracellular signaling by interacting with other cell surface receptors. Previous studies have shown that OPN binds to the CD44 receptor to mediate chondrocyte degeneration and alter the expression of HA-related factors [36, 37]. Third, combined with previous literature studies [20], we speculate that there is a bidirectional regulatory relationship between OPN and HA. Blocking the binding of OPN to integrin receptors unidirectionally will not completely silence HA expression. However, the mechanistic details of this signaling need to be delineated. As a high molecular weight polysaccharide, HA is an important component of synovial fluid and articular cartilage. Intraarticularly injecting HA into the knee joint lubricates the articular surface, reduces wear and tear, nourishes the articular cartilage, and promotes the synthesis of endogenous HA, thereby delaying pathological changes in joint. However, the efficacy of single drug therapy targeting HA in OA has certain limitations [38, 39]. Combinations of HA with glucocorticoid or platelet-rich plasma has been used in the treatment of OA. Titan et al. [40] used platelet-rich concentrate in combination with HA in bovine cartilage to enhance cartilage injury repair. Smith et al. [41] reported that the intraarticular injection comprising corticosteroid and HA exhibited better pain relief than that obtained by injecting only HA. We demonstrated that OPN regulates the expression of HAS by binding with integrin *ανβ*3 and, subsequently, affecting HA secretion. OPN expression positively correlated with HA synthesis and secretion. OPN may be involved in the development and repair of articular cartilage.

## 5. Conclusion

We have demonstrated that OPN interacts with integrin *ανβ*3 to regulate HAS expression and HA secretion in OA chondrocytes. Therefore, OPN may serve as a novel candidate for use in combination with HA for the treatment of OA and help other researchers in the field.

## Figures and Tables

**Table 1:** Primer sequence.

Primer name	Primer sequence
*β*-Actin-forward primer	5′-ggaaatcgtgcgtgacatta-3′
*β*-Actin-reverse primer	5′-ggagcaatgatcttgatcttc-3′
HAS1-forward primer	5′-gcgatactgggtagccttca-3′
HAS1- reverse primer	5′-ggttgtaccaggcctcaaga-3′
HAS2-reverse primer	5′-acagacaggctgaggacgac-3′
HAS2-reverse primer	5′-ctgtgattccaaggaggag-3′
HAS3-forward primer	5′-gtcatgtacacggccttcaa-3′
HAS3-reverse primer	5′-cctacttggggatcctcctc-3′
OPN-forward primer	5′-gtgggaaggacagttatcaa-3′
OPN-reverse primer	5′-ctgactttggaaagttcctg-3′
Integrin *αν*-forward primer	5′-tcggatcaagtggcagaaatc-3′
Integrin *αν*-reverse primer	5′-aaatctccgacagccacag-3′
Integrin *β*3-forward primer	5′-ccctgctcatctggaaactc-3′
Integrin *β*3-reverse primer	5′-cggtacgtgatattggtgaagg-3′

## Data Availability

All data used to support the results of this study are available from the corresponding author upon request.

## Abbreviations

OA: Osteoarthritis
OPN: Osteopontin
HAS: Hyaluronic acid synthases
HA: Hyaluronic acid
RT-qPCR: Real-time fluorescence quantitative polymerase chain reaction
ELISA: Enzyme-linked immunosorbent assay.

## References

[1] Hasegawa M., Segawa T., Maeda M., Yoshida T., Sudo A. (2011). Thrombin-cleaved osteopontin levels in synovial fluid correlate with disease severity of knee osteoarthritis. *The Journal of Rheumatology*.

[2] Shang H., Hao Y., Hu W., Hu X., Jin Q. (2019). *OPN* gene locus is associated with the risk of knee osteoarthritis: a case-control study. *Bioscience Reports*.

[3] Blagojevic M., Jinks C., Jeffery A., Jordan K. P. (2010). Risk factors for onset of osteoarthritis of the knee in older adults: a systematic review and meta-analysis. *Osteoarthritis and Cartilage*.

[4] Bobick B. E., Kulyk W. M. (2008). Regulation of cartilage formation and maturation by mitogen-activated protein kinase signaling. *Birth Defects Research Part C: Embryo Today: Reviews*.

[5] Liang J., Xu L., Zhou F. (2018). MALAT1/miR-127-5p regulates osteopontin (OPN)-mediated proliferation of human chondrocytes through PI3K/Akt pathway. *Journal of Cellular Biochemistry*.

[6] Standal T., Borset M., Sundan A. (2004). Role of osteopontin in adhesion, migration, cell survival and bone remodeling. *Experimental Oncology*.

[7] El-Tanani M. K. (2008). Role of osteopontin in cellular signaling and metastatic phenotype. *Frontiers in Bioscience*.

[8] Gao S. G., Li K. H., Zeng K. B., Tu M., Xu M., Lei G. H. (2010). Elevated osteopontin level of synovial fluid and articular cartilage is associated with disease severity in knee osteoarthritis patients. *Osteoarthritis and Cartilage*.

[9] Li L., Lv G., Wang B., Kuang L. (2020). XIST/miR-376c-5p/OPN axis modulates the influence of proinflammatory M1 macrophages on osteoarthritis chondrocyte apoptosis. *Journal of Cellular Physiology*.

[10] Wang Z., Boyko T., Tran M. C. (2018). DEL1 protects against chondrocyte apoptosis through integrin binding. *The Journal of Surgical Research*.

[11] Wang Q., Onuma K., Liu C. (2019). Dysregulated integrin *α*_V_*β*3 and CD47 signaling promotes joint inflammation, cartilage breakdown, and progression of osteoarthritis. *JCI Insight*.

[12] Tian J., Zhang F. J., Lei G. H. (2015). Role of integrins and their ligands in osteoarthritic cartilage. *Rheumatology International*.

[13] Loeser R. F. (2014). Integrins and chondrocyte-matrix interactions in articular cartilage. *Matrix Biology*.

[14] Ruhlen R., Marberry K. (2014). The chondrocyte primary cilium. *Osteoarthritis and Cartilage*.

[15] Kale S., Raja R., Thorat D., Soundararajan G., Patil T. V., Kundu G. C. (2015). Osteopontin signaling upregulates cyclooxygenase-2 expression in tumor-associated macrophages leading to enhanced angiogenesis and melanoma growth via *α*9*β*1 integrin. *Oncogene*.

[16] Wilson L. A., Liu J., Fiasconaro M., Poeran J., Nwachukwu B. U., Memtsoudis S. G. (2020). Increased use of intra-articular steroid injection to treat osteoarthritis is associated with chronic opioid dependence after later total knee arthroplasty but not total hip arthroplasty. *The Journal of Arthroplasty*.

[17] Nagaoka A., Yoshida H., Nakamura S. (2015). Regulation of hyaluronan (HA) metabolism mediated by HYBID (hyaluronan-binding protein involved in HA depolymerization, KIAA1199) and HA synthases in growth factor-stimulated fibroblasts. *The Journal of Biological Chemistry*.

[18] Törrönen K., Nikunen K., Kärnä R., Tammi M., Tammi R., Rilla K. (2014). Tissue distribution and subcellular localization of hyaluronan synthase isoenzymes. *Histochemistry and Cell Biology*.

[19] Elliott A. L., Kraus V. B., Luta G. (2005). Serum hyaluronan levels and radiographic knee and hip osteoarthritis in African Americans and Caucasians in the Johnston County Osteoarthritis Project. *Arthritis and Rheumatism*.

[20] Zhang F. J., Gao S. G., Cheng L. (2013). The effect of hyaluronic acid on osteopontin and CD44 mRNA of fibroblast-like synoviocytes in patients with osteoarthritis of the knee. *Rheumatology International*.

[21] Liu Q., He H., Yuan Y., Zeng H., Wang Z., Luo W. (2020). Novel expression of EGFL7 in osteosarcoma and sensitivity to cisplatin. *Frontiers in Oncology*.

[22] Qian J., Oppermann E., Tran A., Imlau U., Qian K., Vogl T. J. (2016). Transarterial administration of integrin inhibitor loaded nanoparticles combined with transarterial chemoembolization for treating hepatocellular carcinoma in a rat model. *World Journal of Gastroenterology*.

[23] Georgoulis A., Havaki S., Drosos Y. (2012). RGD binding to integrin alphavbeta3 affects cell motility and adhesion in primary human breast cancer cultures. *Ultrastructural Pathology*.

[24] De Fusco C., Messina A., Monda V. (2017). Osteopontin:relation between adipose tissue and bone homeostasis. *Stem Cells International*.

[25] Clemente N., Raineri D., Cappellano G. (2016). Osteopontin bridging innate and adaptive immunity in autoimmune diseases. *Journal of Immunology Research*.

[26] Shi L., Wang X. (2017). Role of osteopontin in lung cancer evolution and heterogeneity. *Seminars in Cell & Developmental Biology*.

[27] Liu Q., Zeng H., Yuan Y., Wang Z., Wu Z., Luo W. (2020). Osteopontin inhibits osteoarthritis progression via the OPN/CD44/PI3K signal axis. *Genes & Diseases*.

[28] Wang Q., Wang W., Zhang F., Deng Y., Long Z. (2017). NEAT1/miR-181c regulates osteopontin (OPN)-mediated synoviocyte proliferation in osteoarthritis. *Journal of Cellular Biochemistry*.

[29] Sun S. J., Wu C. C., Sheu G. T. (2016). Integrin *β*3 and CD44 levels determine the effects of the OPN-a splicing variant on lung cancer cell growth. *Oncotarget*.

[30] Fan C. S., Chen W. S., Chen L. L. (2018). Osteopontin-integrin engagement induces HIF-1*α*-TCF12-mediated endothelial-mesenchymal transition to exacerbate colorectal cancer. *Oncotarget*.

[31] Guo Y., Xu H., Li Y. (2017). Hyaluronic acid and Arg-Gly-Asp peptide modified graphene oxide with dual receptor-targeting function for cancer therapy. *Journal of Biomaterials Applications*.

[32] Mikuła-Pietrasik J., Sosińska P., Książek K. (2014). Resveratrol inhibits ovarian cancer cell adhesion to peritoneal mesothelium in vitro by modulating the production of *α*5*β*1 integrins and hyaluronic acid. *Gynecologic Oncology*.

[33] Dijkgraaf I., Yim C. B., Franssen G. M. (2011). PET imaging of *α*v*β*₃ integrin expression in tumours with ⁶⁸Ga-labelled mono-, di- and tetrameric RGD peptides. *European Journal of Nuclear Medicine and Molecular Imaging*.

[34] Nikolić I., Stanković N. D., Bicker F. (2013). EGFL7 ligates *α*v*β*3 integrin to enhance vessel formation. *Blood*.

[35] Cao J., Li J., Sun L. (2019). Hypoxia-driven paracrine osteopontin/integrin *α*v*β*3 signaling promotes pancreatic cancer cell epithelial-mesenchymal transition and cancer stem cell-like properties by modulating forkhead box protein M1. *Molecular Oncology*.

[36] Lan T., Pang J., Wu Y. (2016). Cross-linked hyaluronic acid gel inhibits metastasis and growth of gastric and hepatic cancer cells: in vitro and in vivo studies. *Oncotarget*.

[37] Cai Y., López-Ruiz E., Wengel J., Creemers L. B., Howard K. A. (2017). A hyaluronic acid-based hydrogel enabling CD44-mediated chondrocyte binding and gapmer oligonucleotide release for modulation of gene expression in osteoarthritis. *Journal of Controlled Release*.

[38] Annaniemi J. A., Pere J., Giordano S. (2019). Platelet-rich plasma versus hyaluronic acid injections for knee osteoarthritis: a propensity-score analysis. *Scandinavian Journal of Surgery*.

[39] Raeissadat S. A., Rayegani S. M., Hassanabadi H. (2015). Knee osteoarthritis injection choices: platelet- rich plasma (PRP) versus hyaluronic acid (a one-year randomized clinical trial). *Clinical Medicine Insights: Arthritis and Musculoskeletal Disorders*.

[40] Titan A., Schär M., Hutchinson I., Demange M., Chen T., Rodeo S. (2020). Growth factor delivery to a cartilage-cartilage interface using platelet-rich concentrates on a hyaluronic acid scaffold. *Arthroscopy*.

[41] Smith C., Patel R., Vannabouathong C. (2019). Combined intra-articular injection of corticosteroid and hyaluronic acid reduces pain compared to hyaluronic acid alone in the treatment of knee osteoarthritis. *Knee Surgery, Sports Traumatology, Arthroscopy*.

